# Concurrent Immune Suppression and Hyperinflammation in Patients With Community-Acquired Pneumonia

**DOI:** 10.3389/fimmu.2020.00796

**Published:** 2020-05-06

**Authors:** Xanthe Brands, Bastiaan W. Haak, Augustijn M. Klarenbeek, Natasja A. Otto, Daniël R. Faber, René Lutter, Brendon P. Scicluna, W. Joost Wiersinga, Tom van der Poll

**Affiliations:** ^1^Center for Experimental and Molecular Medicine (CEMM), Amsterdam University Medical Centers – Location AMC, University of Amsterdam, Amsterdam, Netherlands; ^2^Department of Internal Medicine, BovenIJ Hospital, Amsterdam, Netherlands; ^3^Respiratory Medicine and Experimental Immunology, Amsterdam University Medical Centers – Location AMC, University of Amsterdam, Amsterdam, Netherlands; ^4^Department of Clinical Epidemiology, Biostatistics and Bioinformatics, Amsterdam University Medical Centers – Location AMC, University of Amsterdam, Amsterdam, Netherlands; ^5^Division of Infectious Diseases, Amsterdam University Medical Centers – Location AMC, University of Amsterdam, Amsterdam, Netherlands

**Keywords:** community-acquired pneumonia, immune suppression, systemic inflammation, sepsis, lipopolysaccharide

## Abstract

**Background:**

The nature and timing of the host immune response during infections remain uncertain and most knowledge is derived from critically ill sepsis patients. We aimed to test the hypothesis that community-acquired pneumonia (CAP) is associated with concurrent immune suppression and systemic inflammation.

**Methods:**

Blood was collected from 79 CAP patients within 24 h after hospitalization and 1 month after discharge; 42 age- and sex-matched subjects without acute infection served as controls. Blood leukocytes were stimulated with lipopolysaccharide (LPS) or *Klebsiella pneumoniae*, and cytokines were measured in supernatants. Fifteen plasma biomarkers reflective of key host response pathways were compared between CAP patients with the strongest immune suppression (lowest 25% blood leukocyte tumor necrosis factor (TNF)-α production in response to LPS) and those with the least immune suppression (highest 25% of LPS-induced TNF-α production).

**Results:**

Blood leukocytes of CAP patients (relative to control subjects) showed a reduced capacity to release TNF-α, interleukin (IL)-1β, IL-6 and IL-10 upon stimulation with LPS or *K. pneumoniae*, with a concurrently enhanced ability to release the anti-inflammatory mediator IL-1 receptor antagonist, irrespective of the presence of sepsis (18.9% of cases). Low (relative to high) TNF-α producers displayed higher plasma levels of biomarkers reflecting systemic inflammation, neutrophil degranulation, endothelial cell activation, a disturbed vascular barrier function and coagulation activation.

**Conclusion:**

CAP replicates a common feature of immune suppression in sepsis. The coexistence of immune suppression and hyperinflammation in CAP argues against the theory of two distinct phases during the host response to sepsis.

## Introduction

Community-acquired pneumonia (CAP) is the world’s most prevalent and deadly infectious disease, responsible for 3 million deaths worldwide in 2016 (1). The annual incidence ranges between 2.7 and 10 per 1000 persons; (2) in the United States the incidence of CAP requiring hospitalization has been estimated at 24.8 cases per 10,000 adults per year, with the highest rates in elderly people (3, 4). CAP is the main cause of sepsis, (5) a life-threatening syndrome characterized by a dysregulation of the host response during infection, which is associated with high morbidity and mortality (6).

The innate immune system is essential for the initiation of a robust inflammatory host response after detecting danger signals from pathogens (7). For many years it was thought that sepsis embodies an uncontrolled pro-inflammatory response driven by the production of pro-inflammatory cytokines such as tumor necrosis factor (TNF)-α and interleukin (IL)-1β in the first hours after infection (8–11). This initial “cytokine storm” was implicated as a main driver of early mortality in infections. More recently, it has become clear that sepsis is also associated with anti-inflammatory responses, characterized amongst others by the production of anti-inflammatory cytokines such as IL-10 and IL-1 receptor antagonist (IL-1RA) and a reduced capacity of blood leukocytes to release proinflammatory cytokines in response to stimulation with lipopolysaccharide (LPS) or other bacterial agonists (7–9, 12, 13). These anti-inflammatory reactions may result in immune suppression and have been associated with secondary infections and late sepsis mortality (7–9, 12, 13).

The immunological aspects of sepsis have been studied predominantly in critically ill patients upon admission to the Intensive Care Unit. Knowledge of the existence and extent of immune suppressive responses in less severe infections is more limited. Such information might provide insight into the severity of the disease required to elicit immune suppressive reactions, as well as into the timing of hyper-inflammatory and immunosuppressive responses, considering the longstanding theory that pro-inflammatory responses may precede anti-inflammatory reactions during the development of sepsis (9, 12, 13). Therefore, we aimed to determine whether CAP patients show signs of immune suppression, and if so, whether these are associated with the presence of sepsis and concurrent pro-inflammatory responses. For this, we used the responsiveness of whole blood leukocytes to LPS as a readout for immune suppression and measured a comprehensive set of plasma biomarkers reflecting a variety of systemic pro-inflammatory responses linked to specific pathophysiological mechanisms.

## Materials and Methods

### Study Population and Sample Collection

This study was part of the ELDER-BIOME project (clinicaltrials.gov identifier NCT02928367) approved by the medical ethical committees of the Academic Medical Center and BovenIJ hospital in Amsterdam. Consecutive patients older than 18 years admitted between October 2016 and July 2018 to the Academic Medical Center or BovenIJ hospital were screened by trained research physicians. Patients were included if they were admitted with an acute infection of the respiratory tract, defined as at least one respiratory symptom (new cough or sputum production, chest pain, dyspnea, tachypnea, abnormal lung examination, or respiratory failure) and one systemic symptom (documented fever or hypothermia, leukocytosis or leukopenia) and had an evident new or progressive infiltrate, consolidation, cavitation, or pleural effusion on chest X ray or computed tomography scan.

Patients were excluded if there was a clinical suspicion of aspiration pneumonia or hospital-associated pneumonia, or if patients were previously diagnosed with malignant hematological disease or exposed to chemotherapy, systemic corticosteroids and/or other immunosuppressive drugs, or if patients were exposed to oral and/or intravenous antibiotics within 48 h prior to hospital admission. Written informed consent was obtained from all eligible participants, or their legal representatives. Ethylenediaminetetraacetic acid (EDTA) blood (for plasma biomarker measurements) and heparin anticoagulated blood (for cell stimulations) was obtained within 24 h of hospital admission and 1 month after admission. Age and sex-matched subjects without acute infection served as controls.

### Clinical Variables

Vital parameters and severity scores, such as the (quick) Sequential Organ Failure Assessment (qSOFA) score (6) and Pneumonia Severity Index (PSI), (14) were calculated upon hospital admission. Systemic inflammatory response syndrome (SIRS) criteria were scored according to the presence of fever >38.0°C or hypothermia <36.0°C; heart rate >90/min; respiratory rate >20 breaths/min; leukocytosis >12 × 10^9^/l or leukocytopenia <4 × 10^9^/l (15). Sepsis was defined according to the Sepsis-3 criteria, defined as a SOFA score of 2 points or more (6). Time to clinical stability in patients was calculated using modified Halm’s criteria, (16) defined as the simultaneous presence of the following clinical parameters for 24 h: temperature ≤ 37.2°C; heart rate ≤100/min; systolic blood pressure >90 mm Hg; respiratory rate ≤ 24/min; SO^2^ ≥ 90% or PaO_2_ ≥ 60 mmHg without supplemental O_2_ or mechanical ventilation.

### Whole Blood Stimulation

Whole blood stimulation was initiated within 2 h after blood sampling in all patients and controls. Heparin-anticoagulated blood was diluted 1:5 in pyrogen free RPMI 1640 (Life Technologies, Bleiswijk, Netherlands), without red blood cell lysis or removal of platelets, and incubated in a cell-repellent surface 48-well plate (Greiner Bio-One, Kremsmünster, Austria) for 24 h at 37°C with 5% CO_2_ and 95% humidity with or without LPS (LPS- *Escherichia coli* 0111:B4 Ultrapure, 100 ng/mL, Invivogen, Toulouse, France), or heat-killed (30 min at 70°C) *Klebsiella pneumoniae* (ATCC43816; equivalent of 12.5 × 10^6^ CFU/mL). After 24 h, samples were centrifuged for 8 min at 1400 revolutions per minute at 4°C and supernatants were stored at −80°C until analysis. Cytokine measurements in supernatants were done in one run, after completion of enrollment, using one batch of assay reagents.

### Assays

To obtain insight into the potential relation between immune suppression and systemic inflammation during CAP and sepsis, we selected and measured 15 biomarkers indicative of activation and/or dysregulation of key host response pathways involved in the pathological process of CAP and sepsis. Systemic inflammation: TNF-α, IL-1β, IL-6, IL-1RA, IL-10, C-reactive protein (CRP), pentraxin-3, soluble triggering receptor expressed on myeloid cells (sTREM)-1, resistin, tenascin-C, trefoil factor (TFF)3; neutrophil degranulation: myeloperoxidase (MPO), proteinase-3, neutrophil gelatinase-associated lipocalin (NGAL); endothelial and procoagulant responses: soluble E-selectin, soluble vascular cell adhesion molecule (sVCAM)-1, angiopoietin-1, angiopoietin-2, D-dimer and protein C. All biomarkers were measured in EDTA anticoagulated plasma using a Luminex multiplex assay (R&D Systems Inc., Minneapolis, MN, United States) and BioPlex 200 (BioRad, Hercules, CA, United States). Leukocyte counts and differentials were determined at hospital admission using routine diagnostic laboratory methods at the study center (analysis on a Sysmex^®^ XN 9000 analyzer (Sysmex Corporation, Kobe, Japan) by fluorescence flow cytometry in K_2_EDTA anticoagulated blood) (17).

### Statistical Analysis and Stratification of CAP Patients Into Low and High TNF-α Producers

Statistical analysis was performed in the R statistical framework (Version 3.51, R Core Team 2014. R: A language and environment for statistical computing. R Foundation for Statistical Computing, Vienna, Austria). All results are individually presented as numbers (percentages) for categorical variables, median and interquartile ranges (IQR, Q1-Q3) for non-parametric quantitative variables, and mean ± standard deviation of the mean (SD) for parametric quantitative variables. Given the premise that a reduced capacity of whole blood leukocytes to produce TNF-α is correlated with immune suppression, (7, 13, 18), we stratified CAP patients into groups of LPS-induced TNF-α production capacity. We selected the patients with the lowest 25% LPS-induced blood leukocyte TNF-α production and compared their host responses with those patients with the highest 25% LPS-induced whole-blood leukocyte TNF-α production. Data distribution was assessed by the Kolmogorov–Smirnov test. Continuous non-parametric data were analyzed using a Mann-Whitney *U* test or a Kruskal–Wallis test; categorical data were analyzed using a χ2 or Fisher exact test. Continuous parametric data were analyzed using a Student *t*-test or analysis of variance. A *p*-value < 0.05 was considered statistically significant. Multiple-comparison adjusted (Benjamini-Hochberg) *P*-value less than 0.05 defined significance of plasma biomarker results (19).

## Results

### Baseline Characteristics and Outcome of Study Population

Between October 2016 and July 2018, 79 patients admitted for CAP and 42 age- and sex-matched controls without infection were included in the study (Table 1). 55 CAP patients were re-evaluated approximately 1 month (mean 33.6 ± SD 5.5 days) after hospital admission. A flowchart of patient inclusion and follow up is depicted in Supplementary Figure S1. Patients admitted with CAP were not different in terms of demographics or chronic comorbidities compared to controls, except for an increased prevalence of chronic obstructive pulmonary disease (COPD) in patients. A causative pathogen was identified in 40 patients (50.4%); a total of 11 patients (13.9%) displayed infection by *Streptococcus pneumoniae* while 7 patients (8.9%) were infected by *Haemophilus influenzae* and 4 by *Staphylococcus aureus* (5,1%). A total of 21 patients (26.6%) were (co)infected with a respiratory virus, of which 10 instances (12,6%) were attributed to Influenza A or Influenza B virus; other prevalent viruses found were rhinovirus (3.8%) and coronavirus (3.8%) (Supplementary Table S1). Of CAP patients, 15 (18.9%) had a SOFA score of 2 or higher. The median time to clinical stability was 4.0 days (IQR 2.0 – 6.0) and median length of hospital stay was 4.5 days (IQR 3.0–8.0). ICU admission occurred in 6 instances (7.6%), 1.0 days (Q1–Q3: 0.0–5.0) following hospital admission; in-hospital and 28-day mortality were 2.5 and 6.5%, respectively.

**TABLE 1:** Baseline characteristics and outcome of patients hospitalized for CAP and controls.

	**CAP**	**Controls**	***P*-value**
Patients, *n*	79	42	
Demographics			
Age, year, mean (SD)	70.5 (13.4)	69.8 (8.41)	0.74
Ethnicity, Caucasian, *n* (%)	60 (74.4)	37 (87.8)	0.20
Body mass index, mean (SD)	26.00 (6.85)	28.07 (5.25)	0.10
Sex, male, *n* (%)	43 (54.4)	22 (52.4)	0.98
Chronic comorbidity, *n* (%)			
COPD	29 (36.7)	4 (9.5)	0.003
Cardiovascular disease	62 (78.5)	26 (61.9)	0.08
Diabetes	20 (25.4)	4 (9.6)	0.09
Malignancy*	22 (27.8)	8 (19.0)	0.40
Neurological disease	7 (8.9)	0 (0.0)	0.11
Gastrointestinal disease	13 (16.5)	2 (4.8)	0.12
Chronic renal disease	6 (7.6)	1 (2.4)	0.45
Severity of disease on admission			
Duration of symptoms prior to admission, days, median [IQR]	4.0 [3.0, 7.0]		
SIRS, median [IQR]	2.0 [1.0, 3.0]		
PSI, median [IQR]	4.0 [3.0, 4.0]		
qSOFA, median [IQR]	1.0 [0.0, 1.0]		
SOFA, median [IQR]	0.0 [0.0, 1.0]		
Sepsis, *n* (%)	15 (19.0)		
Outcome			
ICU admission, *n* (%)	6 (7.6)		
Length of hospital stay, days, median [IQR]	4.5 [3.0, 8.0]		
Time to clinical stability^†^, days, median [IQR]	4.0 [2.0, 6.0]		
Mortality, *n* (%)			
Hospital	2 (2.5)		
28 days	5 (6.3)		

CAP, community-acquired pneumonia; COPD, chronic obstructive pulmonary disease; IQR, interquartile range; SIRS, systemic inflammatory response syndrome; PSI, pneumonia severity index; (q)SOFA, (quick) sequential organ failure assessment; SD, standard deviation of the mean. *Solid malignancy only, not on active chemotherapy. ^†^Calculated according to the modified Halm’s criteria for clinical stability (16).

### Whole Blood Leukocytes of CAP Patients Display Immunosuppressive Responses, Irrespective of the Presence of Sepsis

**FIGURE 1:**
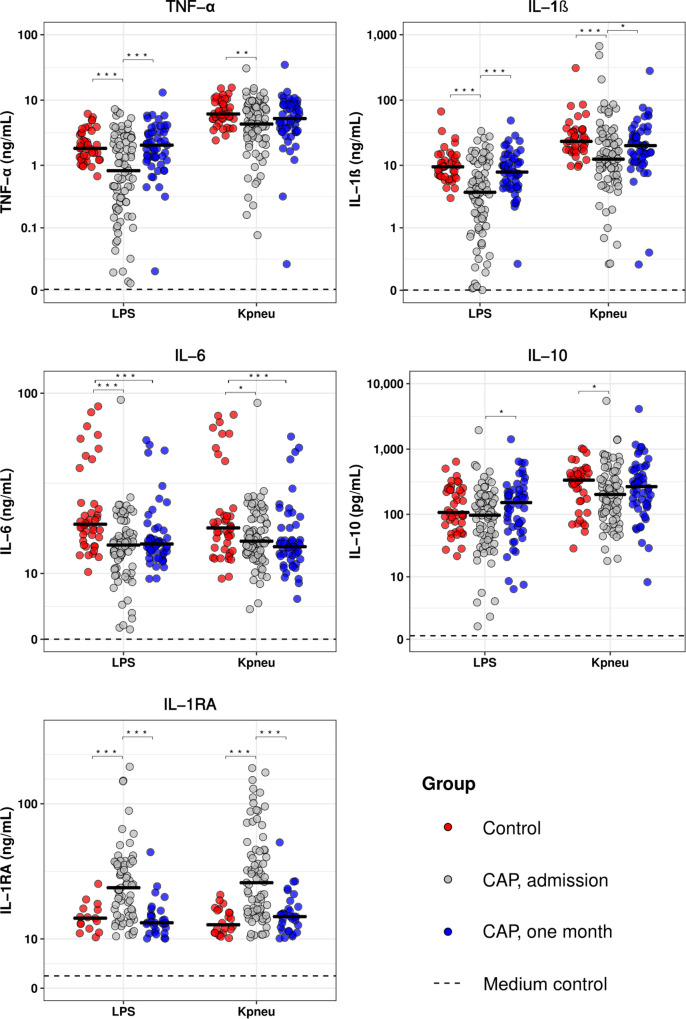
Blood leukocytes of patients with community-acquired pneumonia show an altered cytokine production profile upon *ex vivo* stimulation. Whole blood leukocytes were obtained from CAP patients at admission (*n* = 79) and 1 month following admission (*n* = 55), and from non-infected age and sex-matched controls (*n* = 42), and stimulated for 24 h with lipopolysaccharide (LPS; 100 ng/mL) or heat-killed *Klebsiella pneumoniae* (equivalent of 12.5 × 10^6^ CFU/mL). Cytokines were measured in supernatants. Individual data points are displayed with the horizontal line depicting the median. Dotted lines indicate the median concentrations in medium control samples (i.e., blood leukocytes incubated without stimulus), which were all significantly altered compared to LPS and *K. pneumoniae* stimulation. Asterisks indicate differences between groups as indicated (*P < 0.05, **P < 0.01, ***P < 0.001). IL, interleukin; TNF, tumor necrosis factor; RA, receptor antagonist.

To determine the extent of immune suppression in patients with CAP, we measured the cytokine production capacity of whole blood leukocytes upon stimulation with LPS or *K. pneumoniae* on hospital admission and 1 month thereafter, and compared the results obtained with those from healthy controls (Figure 1). Blood leukocytes obtained from CAP patients at hospital admission displayed a significantly decreased production of TNF-α, IL-1β, IL-6, and IL-10, and enhanced release of IL-1RA following stimulation with LPS or *K. pneumoniae*. Blood leukocytes collected 1 month after CAP admission exhibited similar cytokine production patterns compared to healthy controls, with the exception of IL-6. We subsequently measured 15 plasma markers providing insight in the activation and/or dysregulation of key host response pathways, and observed that CAP patients at hospital admission display elevated levels of host response pathways compared to control subjects and samples collected 1 month following admission (Supplementary Figure S2). Of note, CAP patients without sepsis (SOFA score of 1 or lower, *n* = 64) display similar blood leukocyte responsiveness to LPS and *K. pneumoniae* compared to CAP patients with sepsis (SOFA score of 2 or higher, *n* = 15; Supplementary Figure S3). In addition, CAP patients without sepsis displayed similar levels of plasma host response biomarkers compared to CAP patients with sepsis, with the exception of modestly higher levels of pentraxin-3, resistin, NGAL and sVCAM-1 in CAP patients with sepsis (Supplementary Figure S4). These data show that CAP patients display immunosuppressive responses, irrespective of the presence of sepsis. In addition, 1 month after hospitalization for CAP, whole blood leukocyte responses and plasma protein biomarker levels, at least in part, appear to return to normal homeostasis.

### Stratification of CAP Patients Into Low and High TNF-α Producers

**FIGURE 2:**
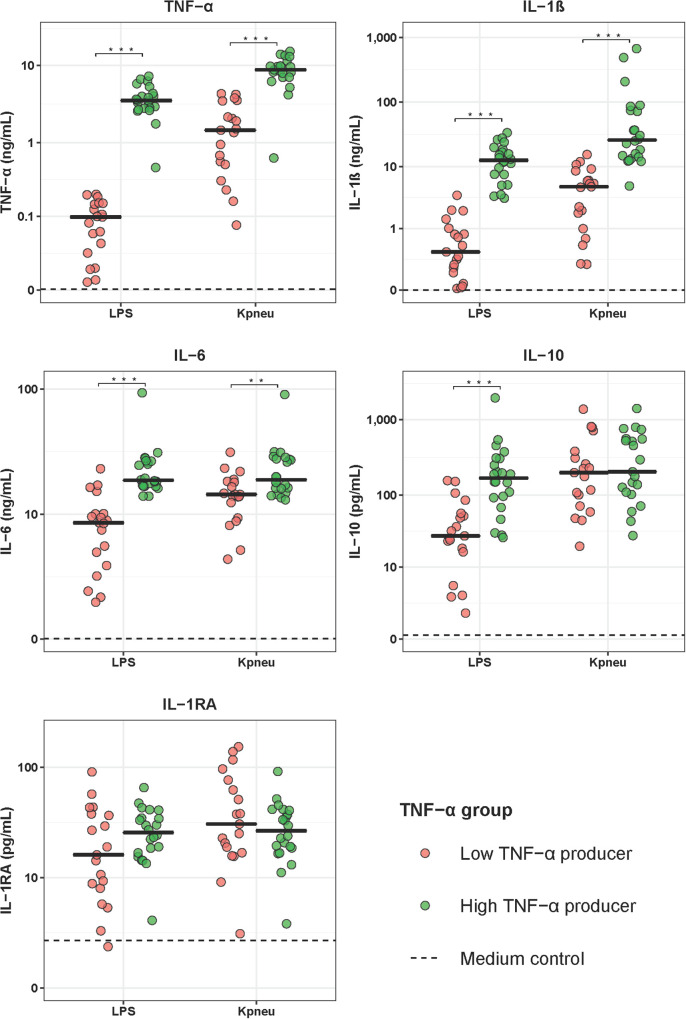
Cytokine production of blood leukocytes from patients with community-acquired pneumonia stratified according to TNF-α production capacity. Patients were stratified into those with the lowest 25% blood leukocyte TNF-α production (low TNF-α producers, *n* = 20) and those with the highest 25% blood leukocyte TNF-α production (high TNF-α producers, *n* = 20) following LPS stimulation. Cytokines were measured in supernatants of whole blood leukocytes stimulated for 24 h with lipopolysaccharide (LPS; 100 ng/mL) or heat-killed *Klebsiella pneumoniae* (equivalent of 12.5 × 10^6^ CFU/mL). Individual data points are displayed with the horizontal line depicting the median. Dotted lines indicate median concentrations in medium control samples (i.e., blood leukocytes incubated without stimulus), which were all significantly altered compared to LPS and *K. pneumoniae* stimulation. Asterisks indicate differences between patients with the lowest and the highest TNF-production following LPS stimulation (**P < 0.01, ***P < 0.001). IL, interleukin; TNF, tumor necrosis factor; RA, receptor antagonist.

To determine if differences in the LPS and *K. pneumoniae* responsiveness of blood leukocytes were associated with altered patterns of systemic inflammation and outcome, we stratified CAP patients into groups of LPS-induced TNF-α production capacity and compared patients with the lowest 25% TNF-α production capacity (low TNF-α producers; most immune suppressed) with those with the highest 25% TNF-α production capacity (high TNF-α producers; least immune suppressed). Low and high TNF-α producers were similar in terms of demographics and prevalence of chronic comorbidities (Table 2). Low TNF-a producers, however, had a higher PSI score and a trend toward a poorer outcome when compared to high TNF-α producers (Table 2). Low (relative to high) TNF-α producers also displayed decreased production of IL-1β and IL-6 following stimulation with LPS or *K. pneumoniae*, which suggests that low TNF-α producers were immune suppressed (Figure 2). White blood cell counts and differentials did not differ between low and high TNF-α producers (Table 3). Likewise, the plasma concentrations of IL-6, IL-8, IL-10, and IL-1RA were not different between groups (Table 3).

**TABLE 2:** Baseline characteristics and outcome in CAP patients with high and low TNF-α production capacity of blood leukocytes.

	**Low TNF-α producers**	**High TNF-α producers**	***P*-value**
Patients, *n*	20	20	
Demographics			
Age, year, mean (SD)	73.6 (10.9)	67.8 (15.6)	0.19
Sex, male, *n* (%)	9 (45.0)	9 (45.0)	>0.99
Ethnicity, Caucasian, *n* (%)	15 (75.0)	17 (85.0)	0.541
Body Mass Index, mean (SD)	25.61 (5.75)	27.33 (7.85)	0.47
Chronic comorbidity, *n* (%)			
COPD	10 (50.0)	4 (20.0)	0.07
Cardiovascular disease	13 (65.0)	16 (80.0)	0.65
Diabetes	5 (25.0)	4 (20.0)	0.71
Malignancy* (%)	5 (25.0)	5 (25.0)	>0.99
Neurological disease (%)	2 (10.0)	1 (5.0)	0.96
Gastrointestinal disease (%)	4 (20.0)	2 (10.0)	0.61
Chronic renal disease	2 (10.0)	2 (10.0)	>0.99
Severity of disease on admission			
Duration of symptoms prior to admission, days, median [IQR]	4.0 [3.0, 6.0]	2.0 [1.0, 5.0]	0.08
SIRS, median [IQR]	2.0 [2.0, 3.0]	2.5 [1.0, 3.0]	0.92
PSI, median [IQR]	4.0 [4.0, 5.0]	3.0 [2.0, 4.0]	0.02
qSOFA, median [IQR]	0.0 [0.0, 1.0]	0.0 [0.0, 1.0]	0.49
SOFA, median [IQR]	0.0 [0.0, 1.0]	0.0 [0.0, 1.0]	0.79
Sepsis, *n* (%)	5 (25.0)	4 (21.1)	1.00
Outcome			
ICU admission, *n* (%)	4 (20.0)	1 (5.0)	0.31
Length of hospital stay, days, median [IQR]	5.0 [3.0, 11.0]	4.0 [2.0, 7.0]	0.48
Time to clinical stability^†^, days, median [IQR]	4.0 [2.0, 8.0]	4.0 [2.0, 6.0]	0.35
Mortality, *n* (%)			
Hospital	1 (5.0)	0 (0.0)	0.79
28 days	2 (10.0)	0 (0.0)	0.47

*COPD, chronic obstructive pulmonary disease; ICU, intensive care unit; IQR, interquartile range; SIRS, systemic inflammatory response syndrome; PSI, pneumonia severity index; (q)SOFA, (quick) sequential organ failure assessment; SD, standard deviation of the mean. ^∗^Solid malignancy only, not on active chemotherapy. ^†^Calculated according to the modified Halm’s criteria for clinical stability (16).*

**TABLE 3:** Plasma cytokine levels, white blood cell counts and differentials in CAP patients with high and low TNF-α production capacity of blood leukocytes.

	**Low TNF-α producers**	**High TNF-α producers**	***P*-value**
White blood cells (×10^9^)	13.10 [9.85 – 18.30]	12.60 [7.6 – 16.8]	0.54
Neutrophils (×10^9^)	10.71 [7.38 – 14.09]	8.34 [4.71 –16.15]	0.76
Monocytes (×10^9^)	0.70 [0.59 – 1.12]	1.14 [0.50 – 1.50]	0.17
Lymphocytes (×10^9^)	0.88 [0.63 –1.14]	1.30 [0.80 – 1.80]	0.12
Platelets (×10^9^)	249 [170.5 – 301]	194 [162 – 240]	0.25
IL-6, pg/mL	85.32 [14.05 – 289.71]	34.27 [26.43 –81.81]	0.14
IL-8, pg/mL	9.51 [5.80 – 18.21]	8.22 [5.54 – 11.32]	0.52
IL-10, pg/mL	8.64 [3.15 – 11.95]	4.11 [2.73 – 5.08]	0.18
IL-1RA, pg/mL	2052 [1355 – 10,902]	3763.9 [1761.8 – 3696.5]	0.73

*All values are presented as a median with the interquartile range in brackets. Definition of abbreviations: IL, interleukin; RA, receptor antagonist.*

### Low TNF-α Producers Show Enhanced Systemic Inflammation and Endothelial-Procoagulant Responses

**FIGURE 3:**
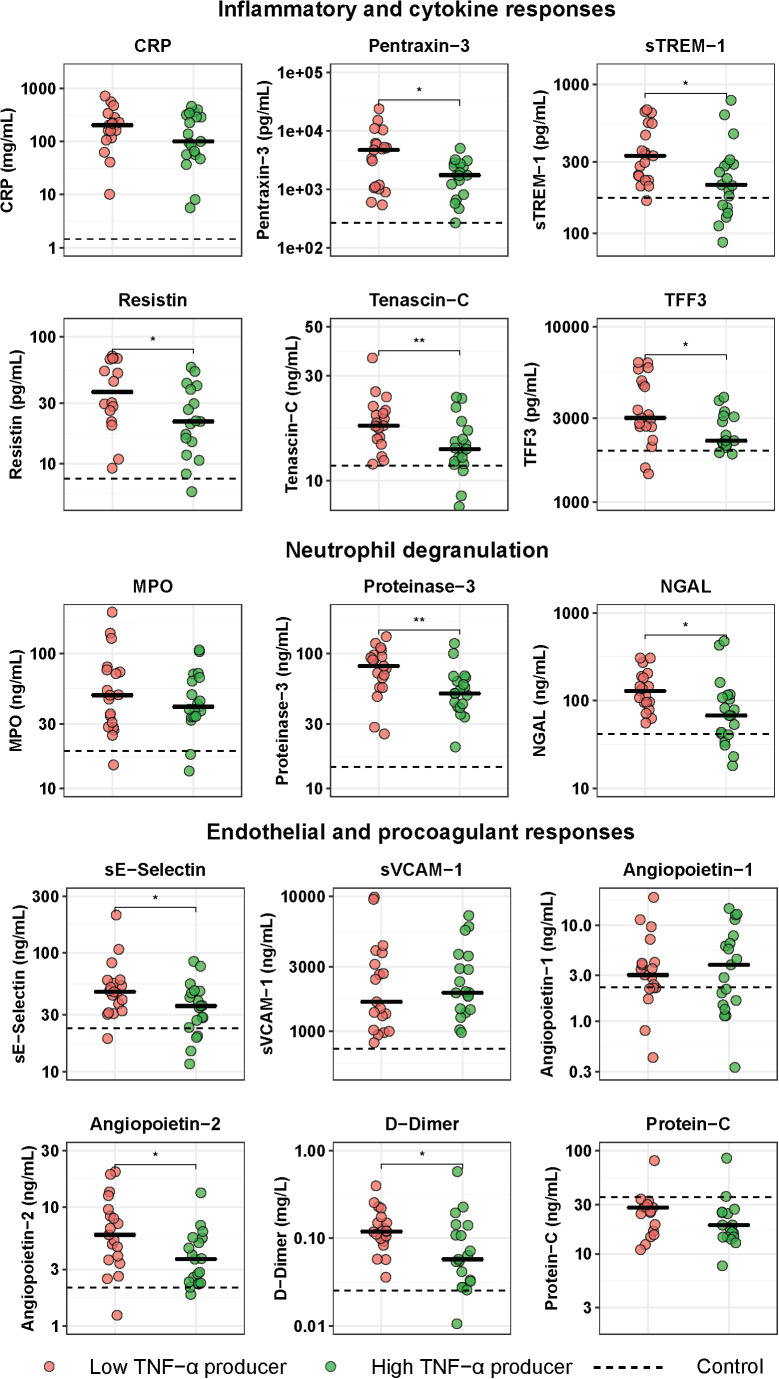
Host response plasma biomarker levels in patients with community-acquired pneumonia with the lowest and highest blood leukocyte TNF-α production following LPS stimulation. Patients were stratified into those with the lowest 25% blood leukocyte TNF-α production (low TNF-α producers, *n* = 20) and those with the highest 25% blood leukocyte TNF-α production (high TNF-α producers, *n* = 20) following LPS stimulation. Plasma biomarkers were measured upon hospital admission. Individual data points are displayed with the horizontal line depicting the median. Dotted lines indicate median values obtained in 42 healthy age- and sex-matched subjects. Values in patients were all significantly different from those in healthy control subjects. Asterisks indicate differences between patients with the lowest 25% and the highest 25% TNF-production following LPS stimulation (Benjamini-Hochberg corrected, *P < 0.05, **P < 0.01). CRP, C-reactive protein; MPO, myeloperoxidase; NGAL, neutrophil gelatinase-associated lipocalin; sE-Selectin, soluble E-selectin; sTREM-1, soluble triggering receptor expressed on myeloid cells 1; sVCAM-1, soluble vascular cell adhesion protein 1; TFF3, trefoil factor 3.

To obtain insight into the potential relation between immune suppression and systemic inflammation, we compared the plasma levels of 15 biomarkers indicative of activation and/or dysregulation of key host response pathways between low and high TNF-α producers (Figure 3). When compared with control subjects, both low and high TNF-α producers exhibited significantly elevated levels of all host response biomarkers on admission to the hospital. Of the biomarkers indicative of systemic inflammation and/or injury, low TNF-α producers had higher plasma levels of pentraxin-3, sTREM-1, resistin, tenascin-C, and TFF3, while CRP tended to be higher in this group, when compared with high TNF-α producers. With regard to plasma biomarkers indicative of neutrophil activation and degranulation, low TNF-α producers showed higher levels of proteinase-3 and NGAL, while MPO concentrations did not differ between groups. With regard to plasma biomarkers indicative of the endothelial and procoagulant responses, low TNF-α producers had higher levels of sE-selectin and D-dimer (reflecting enhanced activation of the endothelium and coagulation system, respectively), and higher levels of angiopoietin-2 (indicative of a more disturbed endothelial barrier function); sVCAM-1, angiopoietin-1 and the anticoagulant protein C did not differ between groups.

## Discussion

A reduced capacity of blood leukocytes to produce proinflammatory cytokines upon *ex vivo* stimulation with bacterial agonists like LPS is considered a main feature of immune suppression in patients with sepsis or non-infectious critical illness (7–9, 12, 13, 18). We here demonstrate that blood leukocytes of hospitalized CAP patients display an impaired release of TNF-α, IL-1β, IL-6, and IL-10 as well as an heightened production of IL-1RA in response to stimulation with LPS and *K. pneumoniae*, regardless of the presence of sepsis. Previous studies reported a similarly modified cytokine profile in LPS-stimulated whole blood in patients with sepsis and septic shock (20–22). These findings indicate that CAP patients display signs of immune suppression at the time of hospital admission. Patients with the most impaired capacity of their blood leukocytes to produce TNF-α concurrently showed heightened inflammatory, endothelial and procoagulant responses. It is well known that CAP patients present with varying degrees of disease severity, ranging from an almost asymptomatic infection to fulminant sepsis with respiratory failure and multiple organ dysfunction (3). It can be speculated that in the absence of adequate (antimicrobial) therapy most CAP cases will evolve into sepsis and that the patients enrolled in our study sought medical attention relatively early. Taken together these data suggest that immune suppression in CAP, as measured by *ex vivo* cytokine production capacity of blood leukocytes, precedes the possible development of sepsis, and that this anti-inflammatory phenotype is accompanied by a variety of systemic proinflammatory responses. Considering that the vast majority of CAP patients did not have sepsis and arguably were studied in a disease phase prior to sepsis development, these results support the by now generally accepted concept of concurrent activation of proinflammatory and anti-inflammatory responses and contradict the old theory of two subsequent phases in the host response in established sepsis with hyperinflammation preceding “compensatory” anti-inflammatory reactions resulting in immune suppression.

Notably, our study entailed a relatively limited number of patients, most of whom did not have sepsis, and the mortality rate was low. The fact that we studied patients with a relatively low disease severity can be considered a strength since virtually all previous investigations that examined immune suppression in infection reported on critically ill patients with sepsis and high mortality rates. In addition, the current selection of patients allowed us to compare differences between patients with and without sepsis, defined according to the most recent sepsis definition. This analysis showed that immune suppression, as reflected by a reduced capacity of blood leukocytes to produce TNF-α, could be detected in sepsis and non-sepsis patients to a similar extent.

One previous investigation studied the capacity of blood leukocytes of ten patients with CAP to release TNF-α in response to LPS stimulation reporting no significant difference when compared with healthy controls (23). This earlier study (23) was limited in sample size (10 patients and 10 controls) and only showed a trend toward reduced TNF-α production by blood leukocytes. In our cohort, blood leukocytes of CAP patients not only showed a reduced capacity to produce prototypic proinflammatory cytokines like TNF-α and IL-1β, but also diminished release of the anti-inflammatory cytokine IL-10. Reduced IL-10 production by blood leukocytes upon re-stimulation has likewise been documented in patients with sepsis (17–19) and healthy humans intravenously injected with LPS, (24, 25) a model frequently used to study mechanisms underlying specific immune responses in sepsis (26, 27). In contrast, severe trauma is associated with enhanced IL-10 release of LPS-stimulated blood leukocytes (28, 29). While differential regulation of IL-10 production is beyond the scope of the present manuscript, it is clear that the alterations in the profile of cytokines released by blood leukocytes of CAP patients resemble that of sepsis patients. This holds true for enhanced IL-1RA release as well, which has been reported in patients with sepsis, (20) and healthy subjects administered with LPS (24, 25).

Epigenetic regulation of gene function, in particular related to histone modifications, has been identified as an important mechanism underlying the altered responsiveness of myeloid cells in patients with sepsis (7). We here show that the capacity of blood leukocytes to produce IL-6 is not yet restored 1 month after admission for CAP. Further studies are warranted to evaluate whether this relates to differences in and/or the duration of epigenetic modifications of IL-6 gene transcription compared to epigenetic modifications of genes encoding TNF-α, IL-1β, IL-10, and IL-1RA, cytokines of which the production by blood leukocytes had returned to control values 1 month after admission for CAP.

In order to determine whether immune suppression associates with concurrent hyperinflammation in CAP, we stratified patients into those with the lowest or highest LPS-induced blood leukocyte production of TNF-α. We confirmed the notion that low TNF-α producers indeed displayed a more profound immunosuppressive phenotype, considering that they also exhibited reductions in IL-1β and IL-6 release, as well as elevated IL-1RA release following LPS or *K. pneumoniae* stimulation, compared to high TNF-α producers. While our study is limited by the fact that we did not measure HLA-DR expression on circulating monocytes, in patients with sepsis a reduced capacity of whole blood leukocytes to produce TNF-α correlated with reduced HLA-DR expression on monocytes, (7, 13, 18) further supporting the concept that this can be used as a readout for immune suppression. We subsequently studied differences in the host response between low and high TNF-α producers amongst CAP patients by using a targeted approach through measuring 15 plasma biomarkers providing insight in key host response pathways implicated in the pathogenesis of severe infections. Low TNF-α producers displayed higher levels of biomarkers associated with inflammation, i.e., pentraxin-3, (30) sTREM-1, (31) tenascin-C, (32) and TFF3, (33). Likewise, low TNF-α producers had higher plasma concentrations of resistin, a mediator linked with exacerbation of inflammation, (34, 35) although more recent evidence has indicated an anti-inflammatory role for resistin in experimental sepsis (36). Another indication that the extent of immune suppression associates with the extent of hyperinflammation in CAP comes from the finding that low TNF-α producers had higher plasma levels of constituents of secondary granules (NGAL) and azurophilic granules (proteinase-3), products of neutrophils known to be highly expressed in response to inflammation (37, 38). Finally, we studied differences between low and high TNF-α producers in endothelial and procoagulant responses, considering their eminent role in sepsis pathogenesis (7, 39, 40). Low TNF-α producers showed signs of stronger endothelial cell and coagulation activation, as reflected by increased sE-selectin and D-dimer levels. In addition, low TNF-α producers had higher angiopoietin-2, but not angiopoietin-1, levels, when compared with high TNF-α producers, which is suggestive of a more disturbed endothelial barrier function in the former, more immune suppressed, group (41).

Previous investigations studied the persistence of immune suppression associated with sepsis after hospital discharge. In 15 septic shock survivors HLA-DR expression on blood monocytes had normalized 6 months after discharge (42). Another study used the plasma concentrations of soluble programmed death ligand 1 (sPD-L1) as a readout for immune suppression and found sustained elevated levels in a subgroup of sepsis survivors up to 1 year after discharge (20). It should be noted, however, that while the PD1-PD-L1 axis has been implicated in sepsis-associated immune suppression, (7, 13) and patients with septic shock displayed increased PD-1 and PD-L1 expression on circulating monocytes and CD4 + T lymphocytes, (43) the plasma levels of sPD-L1 are not necessarily functionally linked to immune suppression. Our study clearly demonstrates that in CAP patients the cytokine production capacity of blood leukocytes is restored to normal 1 month after hospital discharge.

Our study is limited by the fact that we studied the responsiveness of unseparated whole blood leukocytes. Hence, our data do not provide information about the functionality of specific leukocyte subsets, such as T and B cells. To obtain this information cell sorting would be required, which, while of interest, can introduce artifacts due to the strenuous purification procedure.

A strength of this study is the stringent selection of age- and sex-matched control subjects, which was further exemplified by the similarities in comorbidities with CAP patients, with the exception of a lower proportion of COPD in the control group. While chronic inflammation is a hallmark feature of COPD, potentially contributing to immune dysfunction, (44) a previous study has documented enhanced responsiveness of blood leukocytes to LPS (45). In addition, cytokine production reverted to levels comparable to non-infected controls 1 month following admission. Moreover, the main conclusion of our study is derived from patient data only, i.e., comparison between low and high TNF-α producers. Together, these data suggest that the higher prevalence of COPD in patients did not bias our main findings. Despite the strong differences in host response between low and high TNF-α producers, no differences in clinical outcome were found. Low TNF-α producers had a higher PSI indicating that patients who are most immune suppressed have more severe pneumonia; however, this was not reflected by longer hospital stays or significantly higher mortality rates. Notably, this study was not designed to detect such differences, for which a larger sample size would be needed.

This observational study does not provide proof of causal links between immune suppression and hyperinflammation. Nonetheless, our study demonstrates that the host response in CAP patients, irrespective of the presence of sepsis, displays features of both immune suppression (measured as the cytokine production capacity of blood leukocytes) and hyperinflammation (measured by plasma biomarkers reflecting activation of distinct inflammatory and vascular pathways). Considering that most patients did not have sepsis, these results lend support for the theory of simultaneous induction of proinflammatory and anti-inflammatory responses during the host response to sepsis.

## Data Availability Statement

The datasets generated for this study are available on request to the corresponding author.

## Ethics Statement

The studies involving human participants were reviewed and approved by the Medical Ethical Committees of the Academic Medical Center and the BovenIJ hospital. The patients/participants provided their written informed consent to participate in this study.

## Author Contributions

BH and XB conducted data collection, data analysis, data interpretation, and manuscript preparation. AK and NO assisted in data collection and data interpretation. RL oversaw Luminex analysis and assisted in manuscript preparation. DF was the treating physician of included patients and assisted in the manuscript preparation. BS, WW, and TP drafted the study design and data collection protocol, assisted in data analysis and interpretation, and finalized manuscript preparation. All authors critically reviewed and approved the manuscript.

## Conflict of Interest

The authors declare that the research was conducted in the absence of any commercial or financial relationships that could be construed as a potential conflict of interest.

## References

[1] World Health Organization [WHO] *The Top 10 Causes of Death.* (2018). Available online at: http://www.who.int/news-room/fact-sheets/detail/the-top-10-causes-of-death (accessed May 24, 2018)

[2] Schnoor M Hedicke J Dalhoff K Raspe H Schäfer T. Approaches to estimate the population-based incidence of community acquired pneumonia. *J Infect.* (2007) 55:233–9. 10.1016/j.jinf.2007.04.355 17599417

[3] Prina E Ranzani OT Torres A Paulo S Paulo S. Community-acquired pneumonia. *Lancet.* (2015) 386:1097–108. 10.1016/S0140-6736(15)60733-4 26277247 PMC7173092

[4] Jain SS Self WH Wunderink RG Fakhran S Balk R Bramley AM Community-acquired pneumonia requiring hospitalization among U.S, adults. *N Engl J Med.* (2015) 373:415–27. 10.1056/NEJMoa1500245 26172429 PMC4728150

[5] Angus DC van der Poll T. Severe sepsis and septic shock. *N Engl J Med.* (2013) 369:840–51. 10.1056/NEJMra1208623 23984731

[6] Singer M Deutschman CS Seymour CW Shankar-Hari M Annane D Bauer M The third international consensus definitions for sepsis and septic shock (Sepsis-3). *JAMA.* (2016) 315:801 10.1001/jama.2016.0287 PMC4968574 26903338

[7] van der Poll T van de Veerdonk FL Scicluna BP Netea MG. The immunopathology of sepsis and potential therapeutic targets. *Nat Rev Immunol.* (2017) 17:407–20. 10.1038/nri.2017.36 28436424

[8] van der Poll T Opal SM. Host–pathogen interactions in sepsis. *Lancet Infect Dis.* (2008) 8:32–43. 10.1016/S1473-3099(07)70265-7 18063412

[9] Ulloa L Tracey KJ. The ‘cytokine profile”: a code for sepsis.’. *Trends Mol Med.* (2005) 11:56–63. 10.1016/j.molmed.2004.12.007 15694867

[10] Bone RC. Sir Isaac Newton, sepsis, SIRS, and CARS. *Crit Care Med.* (1996) 24:1125–6. 10.1097/00003246-199607000-00010 8674323

[11] Hotchkiss RS Monneret G Payen D. Immunosuppression in sepsis: a novel understanding of the disorder and a new therapeutic approach. *Lancet Infect Dis.* (2013) 13:260–8. 10.1016/S1473-3099(13)70001-X 23427891 PMC3798159

[12] López-Collazo E del Fresno C. Pathophysiology of endotoxin tolerance: mechanisms and clinical consequences. *Crit Care.* (2013) 17:242. 10.1186/cc13110 24229432 PMC4059412

[13] Hotchkiss RS Monneret G Payen D. Sepsis-induced immunosuppression: from cellular dysfunctions to immunotherapy. *Nat Rev Immunol.* (2013) 13:862–74. 10.1038/nri3552 24232462 PMC4077177

[14] Fine MJ Auble TE Yealy DM Hanusa BH Weissfeld LA Singer DE Prediction rule to identify low-risk patients with community-acquired pneumonia. *N Engl J Med.* (1997) 336:243–50. 10.1056/NEJM199701233360402 8995086

[15] Bone RC Balk RA Cerra FB Dellinger RP Fein AM Knaus WA Definitions for sepsis and organ failure and guidelines for the use of innovative therapies in sepsis. The ACCP/SCCM consensus conference committee. american college of chest physicians/society of critical care medicine. *Chest.* (1992) 101:1644–55. 10.1378/chest.101.6.1644 1303622

[16] Halm EA Fine MJ Marrie TJ Coley CM Kapoor WN Obrosky DS Time to clinical stability in patients hospitalized with community-acquired pneumonia. *JAMA.* (1998) 279:1452. 10.1001/jama.279.18.1452 9600479

[17] Schapkaitz E Raburabu S. Performance evaluation of the new measurement channels on the automated Sysmex XN-9000 hematology analyzer. *Clin Biochem.* (2018) 53:132–8. 10.1016/j.clinbiochem.2018.01.014 29374555

[18] Winkler MS Rissiek A Priefler M Schwedhelm E Robbe L Bauer A Human leucocyte antigen (HLA-DR) gene expression is reduced in sepsis and correlates with impaired TNFα response: a diagnostic tool for immunosuppression? *PLoS One.* (2017) 12:e0182427. 10.1371/journal.pone.0182427 28771573 PMC5542660

[19] Hochberg Y Benjamini Y. More powerful procedures for multiple significance testing. *Stat Med.* (1990) 9:811–8. 10.1002/sim.4780090710 2218183

[20] van Deuren M van der Ven Jongekrijg J Demacker PNM Bartelink AKM van Dalen R Sauerwein RW Differential expression of proinflammatory cytokines and their inhibitors during the course of meningococcal infections. *J Infect Dis.* (1994) 169:157–61. 10.1093/infdis/169.1.157 8277177

[21] Nesseler N Martin-Chouly C Perrichet H Ross JT Rousseau C Sinha P Low interleukin-10 release after ex vivo stimulation of whole blood is associated with persistent organ dysfunction in sepsis: a prospective observational study. *Anaesth Crit Care Pain Med.* (2019) 38:485–91. 10.1016/j.accpm.2019.01.009 30797048

[22] Haupt W Zirngibl H Riese J Stehr A Linde HJ Hohenberger W. Depression of tumor necrosis factor-a, interleukin-6, and interleukin-10 production: a reaction to the initial systemic hyperactivation in septic shock. *J Investig Surg.* (1997) 10:349–55. 10.3109/08941939709099598 9654391

[23] De Werra I Zanetti G Faccard C Chioléro R Schaller MD Yersin B CD14 expression on monocytes and TNFα production in patients with septic shock, cardiogenic shock or bacterial pneumonia. *Swiss Med Wkly.* (2001) 131:35–40. 11219189 10.4414/smw.2001.05883

[24] van der Poll T Coyle SM Moldawer LL Lowry SF. Changes in endotoxin-induced cytokine production by whole blood after in vivo exposure of normal humans to endotoxin. *J Infect Dis.* (1996) 174:1356–9. 10.1093/infdis/174.6.1356 8940234

[25] Perlee D van Vught LA Scicluna BP Maag A Lutter R Kemper EM Intravenous infusion of human adipose mesenchymal stem cells modifies the host response to lipopolysaccharide in humans: a randomized, single-blind, parallel group, placebo controlled trial. *Stem Cells.* (2018) 36:1778–88. 10.1002/stem.2891 30063804

[26] Lowry SF. Human endotoxemia: a model for mechanistic insight and therapeutic targeting. *Shock.* (2005) 24:94–100. 10.1097/01.shk.0000191340.23907.a1 16374380

[27] Suffredini AF Noveck RJ. Human endotoxin administration as an experimental model in drug development. *Clin Pharmacol Ther.* (2014) 96:418–22. 10.1038/clpt.2014.146 25236665

[28] Timmermans K Kox M Vaneker M van den Berg M John A van Laarhoven A Plasma levels of danger-associated molecular patterns are associated with immune suppression in trauma patients. *Intensive Care Med.* (2016) 42:551–61. 10.1007/s00134-015-4205-3 26912315 PMC5413532

[29] Adib-Conquy M Moine P Asehnoune K Edouard A Espevik T Miyake K Toll-like receptor-mediated tumor necrosis factor and interleukin-10 production differ during systemic inflammation. *Am J Respir Crit Care Med.* (2003) 168:158–64. 10.1164/rccm.200209-1077OC 12738604

[30] Porte R Davoudian S Asgari F Parente R Mantovani A Garlanda C The long pentraxin PTX3 as a humoral innate immunity functional player and biomarker of infections and sepsis. *Front Immunol.* (2019) 10:794. 10.3389/fimmu.2019.00794 31031772 PMC6473065

[31] Gibot S. Soluble triggering receptor expressed on myeloid cells and the diagnosis of pneumonia and severe sepsis. *Semin Respir Crit Care Med.* (2006) 27:29–33. 10.1055/s-2006-933671 16508879

[32] Yuan W Zhang W Yang X Zhou L Hanghua Z Xu K. Clinical significance and prognosis of serum tenascin-C in patients with sepsis. *BMC Anesthesiol.* (2018) 18:170. 10.1186/s12871-018-0634-1 30442110 PMC6238343

[33] Žurek J Kır M Vavøina M Fedora M. Trefoil factor 3 as a marker of intestinal cell damage during sepsis. *Open Med.* (2015) 10:261–6. 10.1515/med-2015-0020 28352704 PMC5152968

[34] Koch A Gressner OA Sanson E Tacke F Trautwein C. Serum resistin levels in critically ill patients are associated with inflammation, organ dysfunction and metabolism and may predict survival of non-septic patients. *Crit Care.* (2009) 13:R95. 10.1186/cc7925 19545363 PMC2717467

[35] Macdonald SPJ Stone SF Neil CL van Eeden PE Fatovich DM Arendts G Sustained elevation of resistin, NGAL and IL-8 are associated with severe sepsis/septic shock in the emergency department. *PLoS One.* (2014) 9:e110678. 10.1371/journal.pone.0110678 25343379 PMC4208806

[36] Jang JC Li J Gambini L Batugedara HM Sati S Lazar MA Human resistin protects against endotoxic shock by blocking LPS–TLR4 interaction. *Proc Natl Acad Sci USA.* (2017) 114:E10399–408. 10.1073/pnas.1716015114 29133417 PMC5715788

[37] McDonald B. Neutrophils in critical illness. *Cell Tissue Res.* (2018) 371:607–15. 10.1007/s00441-017-2752-3 29247324

[38] Cassatella MA Östberg NK Tamassia N Soehnlein O. Biological roles of neutrophil-derived granule proteins and cytokines. *Trends Immunol.* (2019) 40:648–64. 10.1016/j.it.2019.05.003 31155315

[39] Opal SM van der Poll T. Endothelial barrier dysfunction in septic shock. *J Intern Med.* (2015) 277:277–93. 10.1111/joim.12331 25418337

[40] Levi M van der Poll T. Coagulation and sepsis. *Thromb Res.* (2017) 149:38–44. 10.1016/j.thromres.2016.11.007 27886531

[41] Mikacenic C Hahn WO Price BL Harju-Baker S Katz R Kain KC Biomarkers of endothelial activation are associated with poor outcome in critical illness. *PLoS One.* (2015) 10:e0141251. 10.1371/journal.pone.0141251 26492036 PMC4619633

[42] Zorio V Venet F Delwarde B Floccard B Marcotte G Textoris J Assessment of sepsis-induced immunosuppression at ICU discharge and 6 months after ICU discharge. *Ann Intensive Care.* (2017) 7:80. 10.1186/s13613-017-0304-3 28770544 PMC5540741

[43] Guignant C Lepape A Huang X Kherouf H Denis L Poitevin F Programmed death-1 levels correlate with increased mortality, nosocomial infection and immune dysfunctions in septic shock patients. *Crit Care.* (2011) 15:R99. 10.1186/cc10112 21418617 PMC3219369

[44] Bhat TA Panzica L Kalathil SG Thanavala Y. Immune dysfunction in patients with chronic obstructive pulmonary disease. *Ann Am Thorac Soc.* (2015) 12:S169–75. 10.1513/AnnalsATS.201503-126AW 26595735 PMC4722840

[45] Harting JR Gleason A Romberger DJ Von Essen SG Qiu F Alexis N Chronic obstructive pulmonary disease patients have greater systemic responsiveness to ex vivo stimulation with swine dust extract and its components versus healthy volunteers. *J Toxicol Environ Health.* (2012) 75:1456–70. 10.1080/15287394.2012.722186 23116451 PMC4001714

