# Corrigendum: Concurrent Immune Suppression and Hyperinflammation in Patients With Community-Acquired Pneumonia

**DOI:** 10.3389/fimmu.2020.626667

**Published:** 2020-11-27

**Authors:** Xanthe Brands, Bastiaan W. Haak, Augustijn M. Klarenbeek, Natasja A. Otto, Daniël R. Faber, René Lutter, Brendon P. Scicluna, W. Joost Wiersinga, Tom van der Poll

**Affiliations:** ^1^ Center for Experimental and Molecular Medicine (CEMM), Amsterdam University Medical Centers - Location AMC, University of Amsterdam, Amsterdam, Netherlands; ^2^ Department of Internal Medicine, BovenIJ Hospital, Amsterdam, Netherlands; ^3^ Respiratory Medicine and Experimental Immunology, Amsterdam University Medical Centers - Location AMC, University of Amsterdam, Amsterdam, Netherlands; ^4^ Department of Clinical Epidemiology, Biostatistics and Bioinformatics, Amsterdam University Medical Centers - Location AMC, University of Amsterdam, Amsterdam, Netherlands; ^5^ Division of Infectious Diseases, Amsterdam University Medical Centers - Location AMC, University of Amsterdam, Amsterdam, Netherlands

**Keywords:** community-acquired pneumonia, immune suppression, systemic inflammation, sepsis, lipopolysaccharide

In the original article, there was a mistake in **Figures 1**–**3** as published. The colors in the legend mistakenly mislabeled the conditions. The corrected figures appear below, along with the figure legends, which remain unchanged.

**Figure 1:**
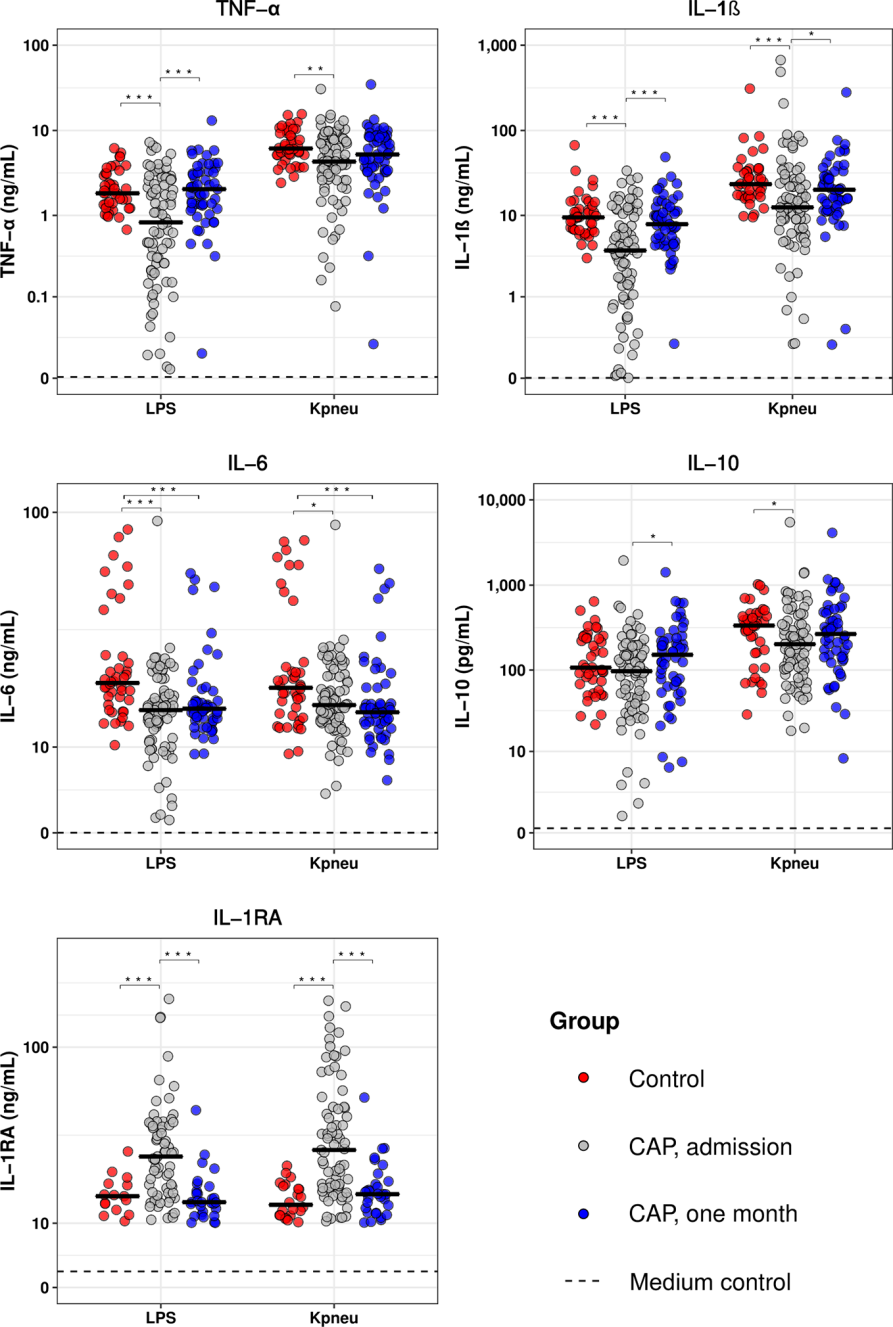
Blood leukocytes of patients with community-acquired pneumonia show an altered cytokine production profile upon *ex vivo* stimulation. Whole blood leukocytes were obtained from CAP patients at admission (n=79) and one month following admission (n=55), and from non-infected age and sex-matched controls (n=42), and stimulated for 24 hours with lipolysaccharide (LPS; 100 ng/mL) or heat-killed *Klebsiella pneumoniae* (Kpneu; equivalent of 12.5 * 10^6^ CFU/mL). Cytokines were measured in supernatants. Individual data points are displayed with the horizontal line depicting the median. Dotted lines indicate the median concentrations in medium control samples (i.e., blood leukocytes incubated without stimulus), which were all significantly altered compared to LPS and Kpneu stimulation. Asterisks indicate differences between groups as indicated (*P < 0.05, **P < 0.01, ***P < 0.001). IL, interleukin; TNF, tumor necrosis factor; RA, receptor antagonist.

**Figure 2:**
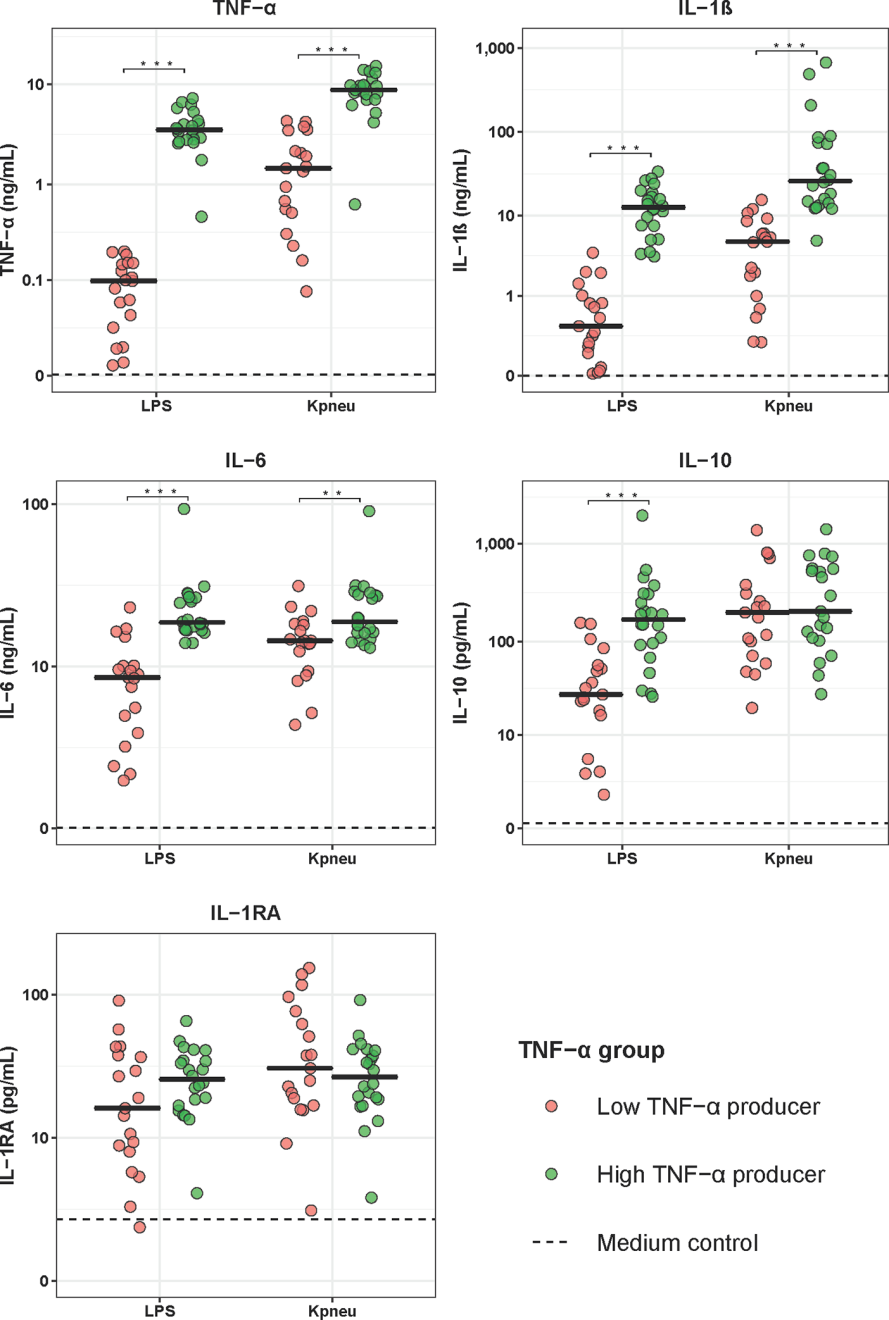
Cytokine production of blood leukocytes from patients with community-acquired pneumonia stratified according to TNF-α production capacity. Patients were stratified into those with the lowest 25% blood leukocyte TNF-α production (low TNF-α producers, n=20) and those with the highest 25% blood leukocyte TNF-α production (high TNF-α producers, n=20) following LPS stimulation. Cytokines were measured in supernatants of whole blood leukocytes stimulated for 24 hours with lipolysaccharide (LPS; 100 ng/mL) or heat-killed *Klebsiella pneumoniae* (Kpneu; equivalent of 12.5 * 10^6^ CFU/mL). Individual data points are displayed with the horizontal line depicting the median. Dotted lines indicate median concentrations in medium control samples (i.e., blood leukocytes incubated without stimulus), which were all significantly altered compared to LPS and Kpneu stimulation. Asterisks indicate differences between patients with the lowest and the highest TNF-production following LPS stimulation (*P < 0.05, **P < 0.01, ***P < 0.001). IL, interleukin; TNF, tumor necrosis factor; RA, receptor antagonist.

**Figure 3:**
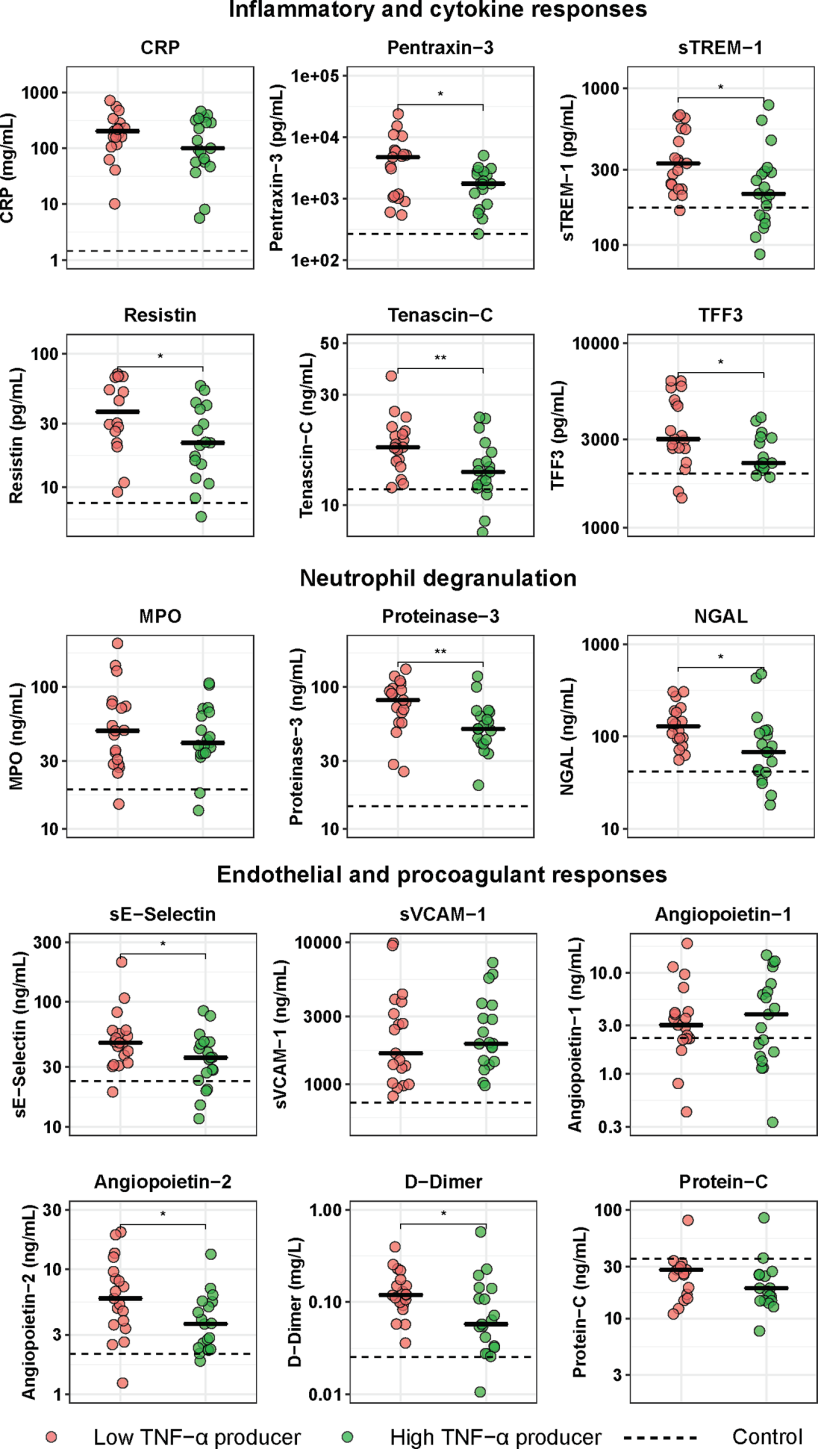
Host response plasma biomarker levels in patients with community-acquired pneumonia with the lowest and highest blood leukocyte TNF-α production following LPS stimulation. Patients were stratified into those with the lowest 25% blood leukocyte TNF-α production (low TNF-α producers, n=20) and those with the highest 25% blood leukocyte TNF-α production (high TNF-α producers, n=20) following LPS stimulation. Plasma biomarkers were measured upon hospital admission. Individual data points are displayed with the horizontal line depicting the median. Dotted lines indicate median values obtained in 42 healthy age- and sex-matched subjects. Values in patients were all significantly different from those in healthy control subjects. Asterisks indicate differences between patients with the lowest 25% and the highest 25% TNF-production following LPS stimulation (Benjamini-Hochberg corrected, *P < 0.05, **P < 0.01). CRP, C-reactive protein; MPO, myeloperoxidase; NGAL, neutrophil gelatinase-associated lipocalin; sE-Selectin, soluble E-selectin; sTREM−1, soluble triggering receptor expressed on myeloid cells 1; sVCAM-1, soluble vascular cell adhesion protein 1; TFF3, trefoil factor 3.

The authors apologize for this error and state that this does not change the scientific conclusions of the article in any way. The original article has been updated.

