# Supplementary material for: Concurrent Immune Suppression and Hyperinflammation in Patients With Community-Acquired Pneumonia
Source: Front Immunol. 2020 May 6;11:796. doi: 10.3389/fimmu.2020.00796 (PMC7232566; doi:10.3389/fimmu.2020.00796)
Supplement: Supplementary file 1 [file Data_Sheet_1.docx]

**SUPPLEMENTAL MATERIAL**

**Concurrent immune suppression and hyperinflammation in patients with community-acquired pneumonia**

Xanthe Brands^1,*^, Bastiaan W. Haak^1,*^, Augustijn M. Klarenbeek^1^, Natasja A. Otto^1^, Daniël R. Faber^2^, René Lutter^3^, Brendon P. Scicluna^1,4^, W. Joost Wiersinga^1,5^, Tom van der Poll^1,5^

^1^Center for Experimental and Molecular Medicine (CEMM), Amsterdam University Medical Centers - Location AMC, University of Amsterdam, Amsterdam, The Netherlands

^2^ Department of Internal Medicine, BovenIJ hospital, Amsterdam, The Netherlands

^3^Respiratory Medicine and Experimental Immunology, Amsterdam University Medical Centers - Location AMC, University of Amsterdam, Amsterdam, The Netherlands

^4^Department of Clinical Epidemiology, Biostatistics and Bioinformatics, Amsterdam University Medical Centers - Location AMC, University of Amsterdam, Amsterdam, The Netherlands

^5^Division of Infectious Diseases, Amsterdam University Medical Centers - Location AMC, University of Amsterdam, Amsterdam, The Netherlands

^*^These authors contributed equally

**Corresponding author:** Xanthe Brands, Center for Experimental and Molecular Medicine (CEMM), Amsterdam University Medical Centers, Location AMC, Room G2-105, Meibergdreef 9, 1105 AZ Amsterdam, The Netherlands; +31 (0)20 5666034; [x.brands@amsterdamumc.nl](mailto:x.brands@amsterdamumc.nl)

**Supplementary Table 1: Causative pathogens identified in cohort***

| **Causative pathogen** | **N (%)** |
| --- | --- |
| *Streptococcus pneumoniae* | 11 (13.6) |
| *Haemophilus influenzae* | 7 (8.8) |
| Influenza A virus | 6 (7.6) |
| Influenza B virus | 4 (5.1) |
| *Staphylococcus aureus* | 4 (5.1) |
| Rhinovirus | 3 (3.8) |
| Coronavirus | 3 (3.8) |
| Respiratory syncytial virus (RSV) | 2 (2.5) |
| Human MetaPneumovirus (hMPV) | 2 (2.5) |
| Parainfluenza virus 1-4 | 1 (1.3) |
| No causative pathogen found | 39 (49.6) |

* Coinfection occurred in 3 instances, percentages exceed 100%

**Supplementary Figure 1: CONSORT Flow diagram of patient inclusion and follow up**

Initial analysis completed (n=79)

- Deceased (n=6)
- Lost to follow up (n=18)

Assessed for eligibility (n=445)

Excluded (n=365)

- No consolidations on imaging (n=152)
- Pneumonia not the primary diagnosis (n=22)
- Aspiration pneumonia (n=18)
- Hospital-acquired Pneumonia (n=28)
- Administration of antibiotics prior to admission (n=54)
- Use of immunosuppressive medication (n=21)
- Haematological malignancy and/or use of chemotherapy (n=14)
- Lacked capacity for informed consent (n=35)
- Eligible, but no informed consent provided (n=21)

Follow up competed (n=55)

No blood leukocyte stimulation data available (n=1)

Included (n=80)

**
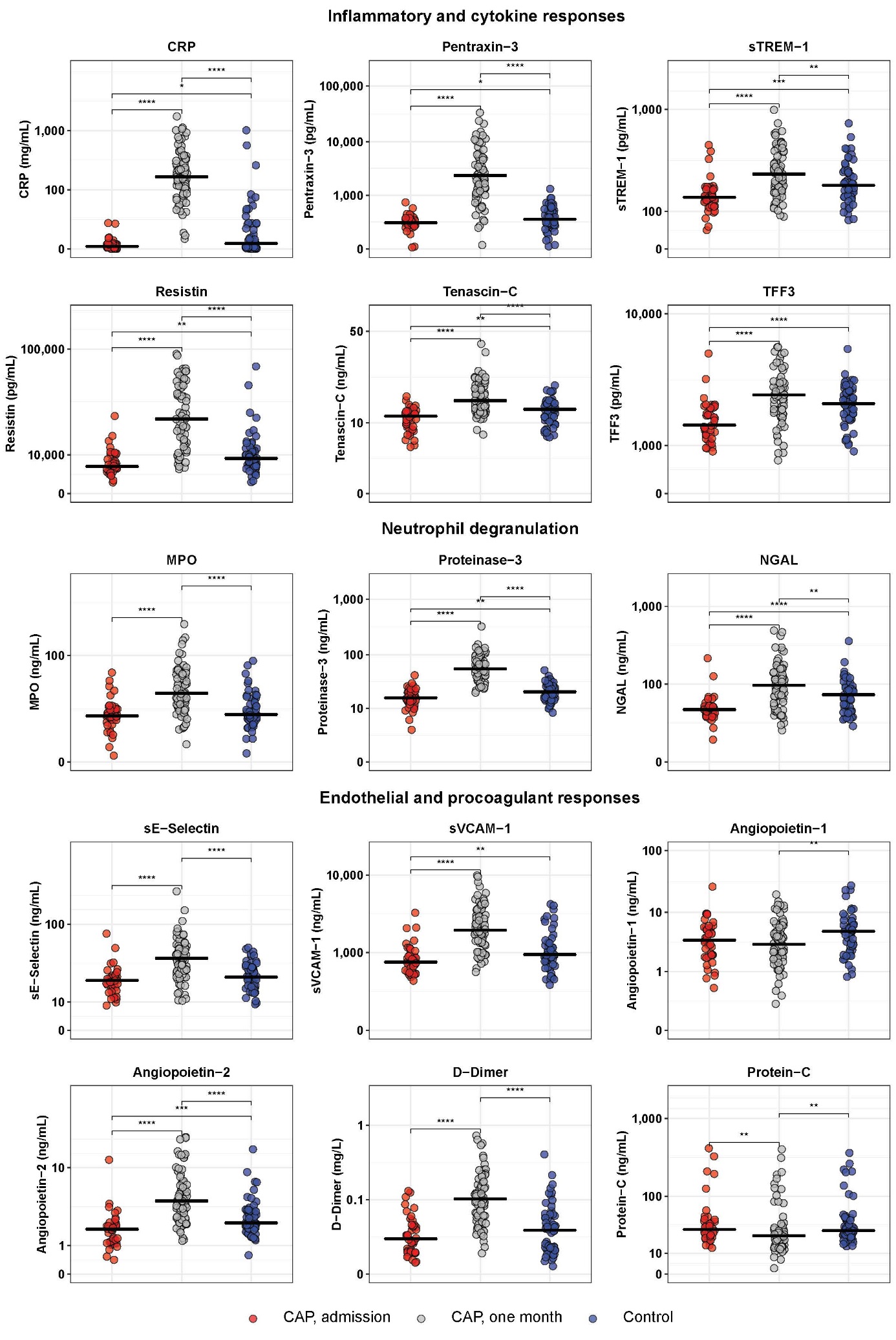
­­­­Supplementary figure 2 Host response plasma biomarker levels in patients with community-acquired pneumonia (at hospital admission and one month thereafter) and control subjects**

**­**Plasma biomarkers were measured in patients upon hospital admission and one month thereafter, as well as in sex-and age-matched controls without acute disease. Individual data points are displayed with the horizontal line depicting the median. (Benjamini-Hochberg corrected, *P<0.05, **P <0.01). CRP = C-reactive protein; MPO = myeloperoxidase; NGAL = neutrophil gelatinase-associated lipocalin; sE-Selectin = soluble E-selectin; sTREM−1 = soluble triggering receptor expressed on myeloid cells 1; sVCAM-1 = soluble vascular cell adhesion protein 1; TFF3 = trefoil factor 3.­­­

**
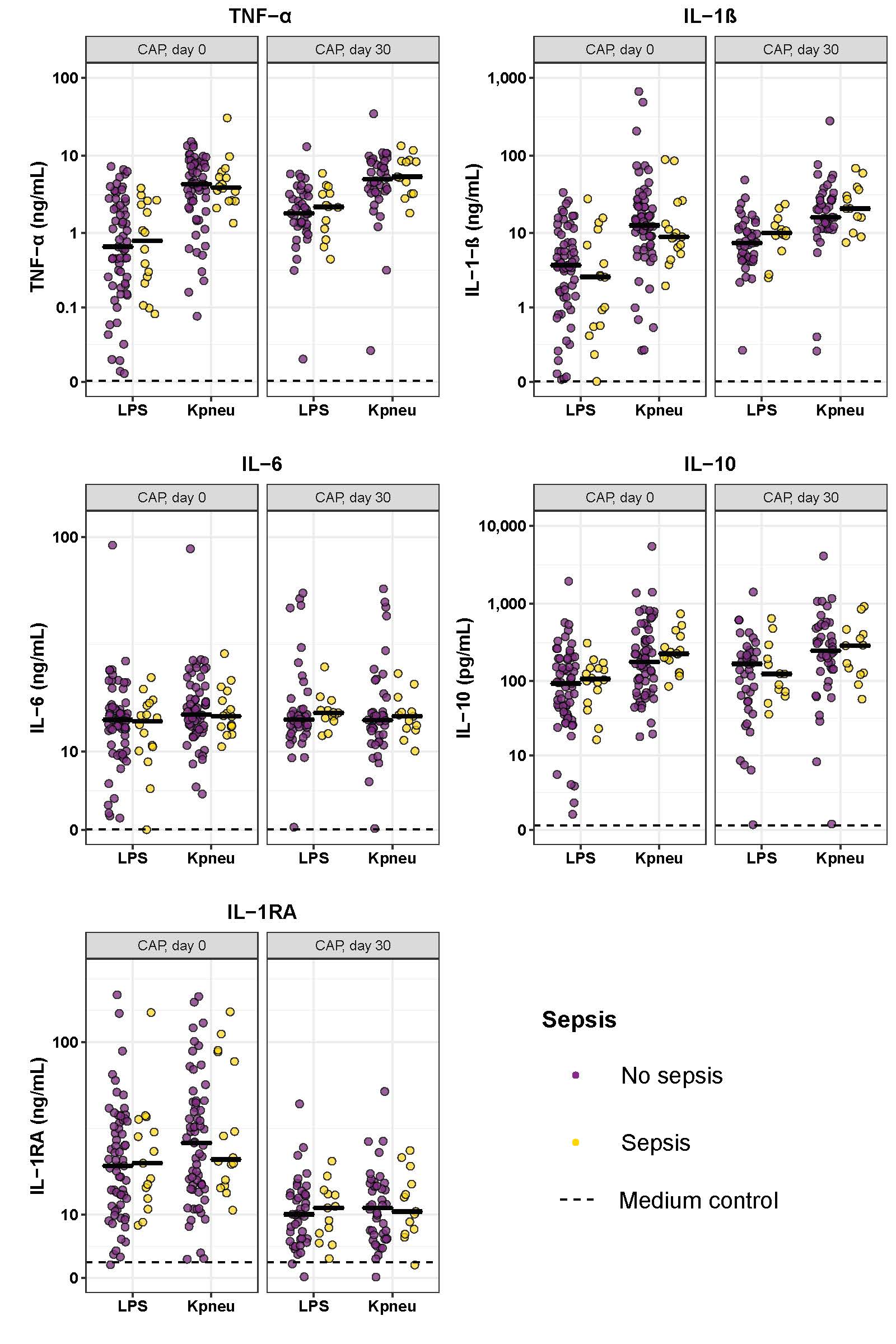
Supplementary figure 3: Whole blood leukocytes of CAP patients without sepsis display similar cytokine production profiles compared to CAP patients with sepsis.**

­­No differences in cytokine production profiles of whole blood leukocytes of CAP patients without sepsis and CAP patients with sepsis, both at admission (n=64 vs n=15, respectively) and one month following admission (n=39 vs n=13, respectively­­) Whole blood leukocytes were stimulated for 24 hours with lipopolysaccharide (LPS; 100 ng/mL) or heat-killed *Klebsiella pneumoniae* (Kpneu; equivalent of 12.5 * 10^6^ CFU/mL). Cytokines were measured in supernatants. Individual data points are displayed with the horizontal line depicting the median. Dotted lines indicate the median concentrations in medium control samples (i.e., blood leukocytes incubated without stimulus), which were all significantly altered compared to LPS and Kpneu stimulation. IL = interleukin; TNF = tumor necrosis factor; RA = receptor antagonist. IL = interleukin; Kpneu = heat-killed *Klebsiella pneumoniae*; LPS = lipopolysaccharide; TNF = tumor necrosis factor; RA = receptor antagonist.

**Supplementary figure 4: Admission plasma host response biomarker levels in CAP patients with or without sepsis.**


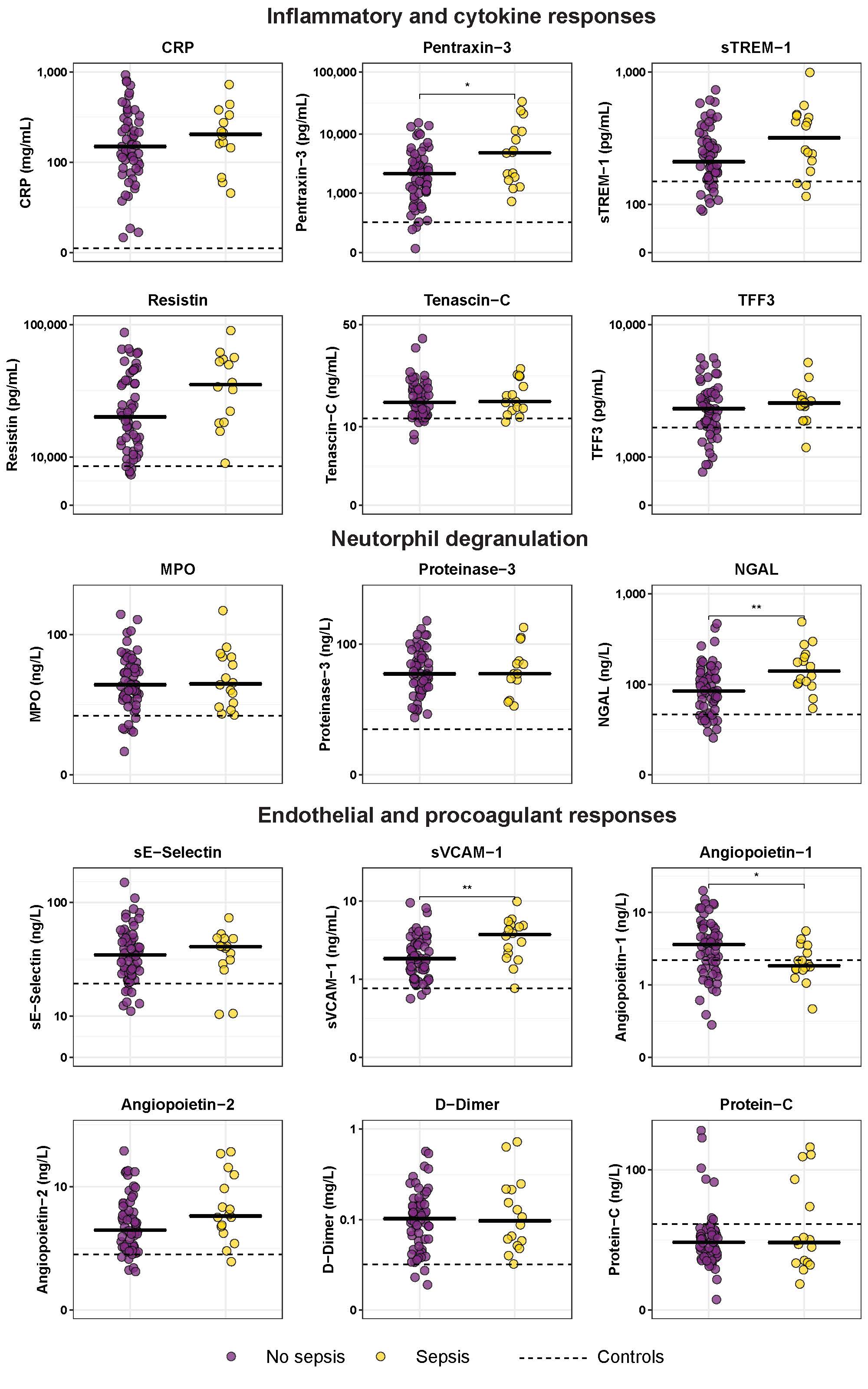


­

Admission plasma biomarkers of CAP patients without sepsis (n=64) and CAP patients with sepsis (n=15). Individual data points are displayed with the horizontal line depicting the median. Dotted lines indicate median values obtained in 42 healthy age- and sex-matched subjects. Values in patients were all significantly different from those in healthy control subjects. Asterisks indicate differences between patients with or without sepsis (Benjamini-Hochberg corrected, *P<0.05, **P <0.01). CRP = C-reactive protein; MPO = myeloperoxidase; NGAL = neutrophil gelatinase-associated lipocalin; sE-Selectin = soluble E-selectin; sTREM−1 = soluble triggering receptor expressed on myeloid cells 1; sVCAM-1 = soluble vascular cell adhesion protein 1; TFF3 = trefoil factor 3.­­­_­­­_
